# Supplementary material for: Molecular physiology of pumiliotoxin sequestration in a poison frog
Source: PLoS One. 2022 Mar 11;17(3):e0264540. doi: 10.1371/journal.pone.0264540 (PMC8916643; doi:10.1371/journal.pone.0264540)
Supplement: Attachment — Submitted filename: response_to_reviewers.docx [file pone.0264540.s002.docx]

**We thank the reviewers for their thoughtful comments on our manuscript. We have addressed each of the comments below.**

**Reviewer 1:**

*1. Correct the statement, “within the poison frog clade, the ability to sequester dietary alkaloids has evolved independently at least three times” (lines 58–59)—the two papers cited to support this statement only show that bright color and diet specialization have independently evolved but do not get at sequestration ability. Instead say something like, “defensive alkaloids have originated at least three times.”*

**Response:** We agree that the above statement does not reflect the papers cited, and have corrected it to read “Within the poison frog clade, defensive alkaloids have originated at least three times.”

*2. It is worth also citing the Daly et al. (2003) PNAS paper (Evidence for an enantioselective pumiliotoxin 7-hydroxylase in dendrobatid poison frogs of the genus Dendrobates [see their figure 3], reference 19) for the assertion that accumulation of alkaloids takes just a few days (line 64).*

**Response:** We agree and have added this citation in line 60 (previously line 64).

*3. PTXs in poison frogs are also known from mites, not only ants as you imply in line 76 (see Saporito et al. [2007] in PNAS, Oribatid mites as a major dietary source for alkaloids in poison frogs, Saporito et al. [2011] in J Chem Ecol, Alkaloids in the mite Scheloribates laevigatus: further alkaloids common to oribatid mites and poison frogs, and Takada et al. [2005] in J Chem Ecol, Scheloribatid mites as the source of pumiliotoxins in dendrobatid frogs).*

**Response:** We apologize for missing these important references and have included them in line 70. We also changed the text of lines 68-70 to “Alkaloids of the pumiliotoxin (PTX) family are found across the poison frogs clade, including *Dendrobates, Oophaga, Epipedobates, Ameerega*, and *Phyllobates* species, and are thought to be sourced from formicine ants and oribatid mites.”

*4. The citation given in line 82 (reference 10) is almost certainly incorrect. Should be Daly et al. (2003) (reference 19).*

**Response:** Thank you for catching this error, it has been corrected.

*5. Line 100 again needs to cite reference 19 and make it clear that the experimental results on aPTX 267A toxicity are also from this study.*

**Response:** This citation has been added as well.

**Reviewer 2:**

*I enjoyed reading this excellent manuscript. This is an elegant study targeting a very relevant question in the evolution of amphibian toxicity, and presents a series of gene expression and protein assay experiments that provide important new insights into the mechanism that underlies biomodification of alkaloids by dendrobatid frogs.*

*The manuscript is excellently written, with basically no typo, and my review therefore is much shorter than usual, as I have almost nothing to criticize. The following are just a few minor points that the author should consider in the revision of the manuscript. I look forward to seeing this great study published!*

**Response:** Thank you for your positive evaluation of our manuscript! We agree that this study provides a step forward in understanding alkaloid metabolism in poison frogs and hope that it will lead to further exploration in this area.

*Line 176 and elsewhere: the tissue type designation "eggs" is misleading; I very much suppose what is meant here is oocytes and not mature eggs. Please change terminology throughout, including in the figures.*

**Response:** Thank you for this suggestion, we agree that the term oocytes is more appropriate given that they were not mature eggs. This has been corrected throughout the manuscript.

*Line 76: Oribatid mites are at least a second, if not the major source of pumiliotoxins besides ants; see Takada et al. doi: 10.1007/s10886-005-7109-9 as well as Saporito et al.* [*https://doi.org/10.1073/pnas.0702851104*](https://doi.org/10.1073/pnas.0702851104) *and other papers.*

**Response:** We apologize for missing these important references and have included them in line 70. We also changed the text of lines 68-70 to “Alkaloids of the pumiliotoxin (PTX) family are found across the poison frogs clade, including *Dendrobates, Oophaga, Epipedobates, Ameerega*, and *Phyllobates* species, and are thought to be sourced from formicine ants and oribatid mites.”

*The list of references requires attention. I guess it was made with a reference managing system which of course is fine but has the drawback of formatting errors. For instance, scientific names are not italicized, and in numerous references, all words are in capitals while they should not be (I noticed the latter at least in refs # 17, 24, 38, 50 but I may have overlooked some).*

**Response:** Thank you for catching these errors, they have been corrected.

*SRA accession numbers need to be added before final publication.*

**Response:** These have been included, along with a data dryad accession for the raw LC-MS/MS data. The text on lines 430-434 now reads “All LC-MS/MS data from the alkaloid analysis, the *D. tinctorius* transcriptome, TMM expression and count data for different tissues, fasta files with nucleotide and amino acid sequences of CYP2D6-like proteins, and code is either available as supplementary materials or are available on the Dryad Digital Repository (https://doi.org/10.5061/dryad.ns1rn8pr3). All Illumina fastq files are available on the Sequence Read Archive (<https://www.ncbi.nlm.nih.gov/sra>, SUB11006206).”
